# *Salmonella*—how a metabolic generalist adopts an intracellular lifestyle during infection

**DOI:** 10.3389/fcimb.2014.00191

**Published:** 2015-01-29

**Authors:** Thomas Dandekar, Astrid Fieselmann, Eva Fischer, Jasmin Popp, Michael Hensel, Janina Noster

**Affiliations:** ^1^Department of Bioinformatics, Biocenter, University of WürzburgWürzburg, Germany; ^2^Division of Microbiology, Biology/Chemistry, University of OsnabrückOsnabrück, Germany

**Keywords:** metabolism, *Salmonella*-containing vacuole (SCV), regulation, virulence, “-omics”

## Abstract

The human-pathogenic bacterium *Salmonella enterica* adjusts and adapts to different environments while attempting colonization. In the course of infection nutrient availabilities change drastically. New techniques, “-omics” data and subsequent integration by systems biology improve our understanding of these changes. We review changes in metabolism focusing on amino acid and carbohydrate metabolism. Furthermore, the adaptation process is associated with the activation of genes of the *Salmonella* pathogenicity islands (SPIs). Anti-infective strategies have to take these insights into account and include metabolic and other strategies. *Salmonella* infections will remain a challenge for infection biology.

## Introduction

*Salmonella enterica* is a Gram-negative enterobacterium closely related to *Escherichia coli* (Neidhardt, 1996). *Salmonellae* reside in humans, a range of animals as well as in the environment and hence are facultative pathogens, often taken up by contaminated food and causing self-limited gastrointestinal disease. In weakened conditions the non-typhoidal serovars may lead to severe bloodstream infections, with high fatality rates in developing countries (Feasey et al., 2012) while typhoidal forms (*S. enterica* serovars Typhi, Paratyphi) strike with endotoxins, typhoid fever, and severe systemic illness. The millions of infections and thousands of fatal cases every year are an important reason for a better understanding and control of *Salmonella* infection (Feasey et al., 2012). To capture the diversity of the *Salmonella* lifestyle in infection is a challenging task. In this review, we will focus on metabolic aspects as well as on insights from “-omics” data, systems biology, and new technologies studying *Salmonella* infection. *Salmonella*, like several other Gamma-proteobacteria, are found in various environments including soils, water systems, and sewage, as well as in the gut flora of various animals. To survive and multiply in this large variety of environments, their metabolism has to adapt well (Rosenkrantz et al., 2013). The large genome of *Salmonella* contains more than 4000 genes encoding a large range of metabolic pathways, for instance an *S*. Typhi chromosome comprises 4,809,037 bp corresponding to 4599 ORFs (including 204 pseudogenes; Parkhill et al., 2001). The pseudogene complement of *S*. Typhi is involved in the tight host restriction of this important human pathogen. There is no zoonotic reservoire. The *S*. Typhi genome reveals an unexpectedly large diversity compared to its relatives *E. coli* and non-typhoidal *Salmonella*.

The lifestyle of *Salmonella*, featuring intestinal colonization, environmental survival, and transmission is reflected in unique gene clusters for adaptation to environmental niches and pathogenicity such as inside the host cell the *Salmonella*-containing vacuole (SCV). Adaptations include multiple abilities for oxygen and nitrate respiration (Rowley et al., 2012). Many further substrates can be used in multiple pathways, depending on environmental conditions. As a food-borne pathogen, various sugars such as D-glucosaminate can be used, supported by suitable permeases (Miller et al., 2013). A vivid picture emerges from data gained by recently established methodologies. Still, not enough is known about regulatory networks around the *Salmonella* Pathogenicity Island, the impact of effector proteins and transport processes and their role in shaping the conditions in the SCV.

In the following we will present established and new approaches of studying *Salmonella* infections, after which we address new perspectives on systems biology including postgenomic modeling techniques and functional genomics. We next discuss stress conditions and specific nutrient supplies and their impact on *Salmonella* metabolism, in particular amino acids and carbohydrate metabolism. Furthermore, connections between metabolism and virulence are discussed. These include SPI1 and SPI2 inducing conditions and their interplay with metabolism. New anti-infective *Salmonella* strategies take these aspects into account. In particular, one has to refine metabolic targeting and drug strategies accordingly. *Salmonella* infection is a particular challenging aspect of its versatile, highly adaptive life style.

### Techniques for studying the intracellular lifestyle of *Salmonella*

Systems biology provides a new technological perspective on *Salmonella* metabolism and virulence: this includes scarless mutation techniques, metabolic flux measurements by isotopologs and sophisticated -omics techniques allowing to study all aspects of the intracellular lifestyle of *Salmonella* in unprecedented detail.

#### Genetics

The very first step in the analysis of the importance of different metabolic enzymes is the generation of mutant strains. For *Salmonella*, the preferred method to rapidly delete chromosomal genes is the phage λ Red deletion technique (Datsenko and Wanner, 2000). Defined single or multiple gene deletion collections for *S*. Typhimurium have recently been published, covering deletions of 3517 genes (Porwollik et al., 2014). Double or multiple mutations, often needed to delete all isoenzymes of a given metabolic pathway, are commonly generated by repeated rounds of Red deletion, combined with phage P22 transduction (Zinder and Lederberg, 1952) and curing of antibiotic resistance. Since the sequential mutagenesis may lead to accumulation of recombination scars and generation of genomic chimera, newer approaches are based on scarless Red recombinase-mediated deletion (Blank et al., 2011).

#### Phenotyping

Before testing the influence of a deactivated metabolic enzyme on *Salmonella* virulence, a primary phenotypic characterization is often performed via determination of growth kinetics. By using minimal medium with different C-sources, Paterson et al. could reveal the ability of a Tpi (triosephosphate-isomerase) deficient strain to utilize gluconate, but not other sources such as glucose (Paterson et al., 2009). Additionally, one can perform growth kinetics with media which mimic different *in vivo* conditions. For instance Wallrodt et al. studied the role of the sulfurtransferases GlpE and PspE for resistance against NO radicals via growth kinetics in minimal medium with S-nitrosoglutathion supplementation (Wallrodt et al., 2013). To investigate the adaptation of *Salmonella* to life within the SCV, conditions inducing SPI2 genes are frequently used, such as minimal medium with low phosphate concentrations (Deiwick et al., 1999).

After these first phenotypic characterizations, the impact of defined gene deletions on *Salmonella* virulence is tested most commonly in cell culture experiments, such as gentamicin protection assays, which provide first clues about the role of metabolic enzymes, transporters, etc., on virulence. In this kind of assays the inability of gentamicin to penetrate into eukaryotic cells is used to kill extracellular bacteria, whereas internalized bacteria do not come into contact with the antibiotic substance (Lobo, 1973). With this method not only *Salmonella's* ability to enter host cells by invasion or phagocytosis but also the intracellular replication ability can be examined (Hölzer and Hensel, 2012).

#### Animal models

Comprehensive *Salmonella* infection models are animals and specific mouse strains are often used. In mice, *Salmonella enterica* serovars pathogenic for humans have been reported (Mathur et al., 2012) not to cause any disease due to an additional Toll-like receptor in mice (TLR11) but further studies have to further confirm this. However, *S. enterica* serovar Typhimurium, which can cause human diarrhea, causes a systemic infection in mice with pathology and disease progression similar to human typhoid fever in mice defective in *Slc11a1* (or *NRAMP*) encoding a Fe^2+^/Mn^2+^/Zn^2+^transporter. Thus, to study the mechanisms of systemic disease caused by *Salmonella*, infection models using *Salmonella*-susceptible inbred mouse strains such as BALB/c or C57BL/6 with defective *Slc11a1* allele are frequently used (Steeb et al., 2013). To understand gastroenteritis caused by *Salmonella*, a major breakthrough was the advent of the Streptomycin-pretreated mouse model. Application of Streptomycin reduces the intestinal microbiota and renders mice susceptible to *Salmonella*-induced intestinal inflammation. For this, C57BL/6 or similar mouse laboratory strains can be used and the *Salmonella* have to be Streptomycin resistant, e.g., *S. enterica* serovar Typhimurium SL1344 (reviewed in Kaiser et al., 2012).

#### Genomics

Methods useful in analyzing the global impact of gene deletions on *Salmonella* and “-omics” techniques (genomics, proteomics, transcriptomics, metabolomics) facilitate studies on virulence mechanisms and metabolic activities on a molecular level and allow a detailed picture of host-pathogen interactions. Comparative genomics was used for example to identify the presence of different metabolic pathways for non-typhoidal and typhoidal pathovars of *Salmonella* (Nuccio et al., 2014). Several recent studies use next generation sequencing (NGS) to understand non-typhoidal *Salmonella* genomes (reviewed by Wain et al., 2013). A broad collection of African isolates showed that they share a common ancestry with *S*. Typhimurium ST313. The study furthermore implies antibiotic resistances were acquired independently in two lineages of *S*. Typhimurium. These data are complemented by phage typing and pulse field gel electrophoresis (PFGE) for additional high resolution typing of *Salmonella* isolates by phage types and different PFGE patterns. This allows investigation in unprecedented detail of virulent strains as well as their correlation with metabolic resistance features such as pathways for degradation of antibiotics.

#### Transcriptomics

The second “-omics” level, namely transcriptomics including microarrays and high throughput sequencing approaches, gives insights into how *Salmonella* regulates its metabolic pathways in response to changing nutritional environments. A study performed by Blair et al. focused on changes in transcriptomic profiles when using LB or various minimal media for growth. Transcription profiles were established and the article instructively starts from microarray experiments (pan-*Salmonella* generation IV microarray) and verifies putative differences by quantitative real-time PCR (Blair et al., 2013). RNA sequencing was applied by Shah (2014) in a recent comparative study of global transcriptomes of high and low pathogenicity (LP) *S. enterica* serovar Enteritidis strains. This technique reveals important links between metabolism and virulence: in LP strains, reduced expression of virulence genes in SPI1 and SPI5 and defensive virulence factors were observed. Interestingly, this was combined with down regulation of metabolic defense pathways, in particular osmotic (glycine betaine/choline transport), oxidative (*katE*, *sodC*), and iron-limiting metabolic protection. In the four ferritins, bacterioferritin (Bfr) was found to be down-regulated in LP strains.

#### Proteomics and metabolomics

Mass spectroscopy (MS)-based proteomics is a method of choice when analyzing gene products: with this approach protein expression is directly measured. Typically only several matching peptides from a protein are identified applying the knowledge of the genome sequence and identified reading frames. This only partial peptide coverage for a given *Salmonella* protein is a challenge for MS analyses. Nevertheless, with more effort even quantification of proteins is possible applying different labeling techniques and standards. A good example for the application of the technique to *Salmonella* is the enzyme quantifications of *ex vivo* purified *Salmonella* performed by Steeb et al. (2013), also illustrating that many proteins can be fast analyzed in this way.

Metabolomics is an upcoming technique as it provides at the same time a global as well as direct view on *Salmonella* metabolism. In particular, isotopolog profiling (IP) allows analysis of current metabolic fluxes under defined conditions. For a detailed method explanation see the study by Härtel et al. (2012), demonstrating the technique on the central carbon metabolism and how individual fluxes are deduced by isotopolog patterns. Furthermore, Götz et al. used this technique to analyze the carbon metabolism of enterobacteria infecting CaCo cells and analyzed which carbon sources are used during intracellular growth (Götz et al., 2010). Metabolic measurements have also been improved by other new techniques such as engineering genetically encoded nanosensors from citrate binding proteins such as the histidine sensor kinase CitA to achieve *in vivo* measurements of changing citrate concentrations in *E. coli* by FRET. This system is readily applicable to *Salmonella* (Ewald et al., 2011).

In general, imaging techniques promote and complement the above approaches to studying the intracellular lifestyle of *Salmonella*. Non-invasive imaging techniques like radioisotope-labeled nucleosides, bioluminescence or the use of microscopy (e.g., advanced light microscopy such as with polarized light) coupled to different cell culture techniques (including establishing tissue infection models) offer here a wealth of information. A nice example including bioluminescent *Salmonella*, the Streptomycin mouse model and bioimaging is Pontier-Bres et al. (2014). Here metabolism and virulence are investigated on possibly the highest level: the protective effect of a pro-biotic food, *Saccharomyces boulardii* and its effect on *Salmonella* clearance in mice.

### “-Omics” data integration and systems biology for studying *Salmonella* during infection

#### Data repositories

The combination of the various “-omics” approaches provides an integrated view on the adaptation of a pathogen to its host, ranging of from understanding of the genetic basis of virulence to the control of metabolic functions within a host organism or host cell. To describe infection processes on a holistic level, multi-omics strategies are required. Large “-omics” datasets on pathogens have become more readily available and have until now shaped the vivid picture of *Salmonella* infection. We present resources of “-omics” data which can be used to integrate and study different levels of systems biology of *Salmonella* infection (Table 1). This list compiles several useful resources but it is of course not exhaustive. Many “-omics” studies rely on large-scale sequence analysis using next-generation sequencing techniques on the genome or on RNA (RNAseq). This includes genome information from the Venter institute, different transcriptome data on gene expression and miRNAs from the Gene Expression Omnibus databank (GEO), proteomics data on membrane proteins from TU Munich, a *Salmonella* wiki on genome information as well as links for veterinary and medical resources on *Salmonella* infection.

**Table 1:** **Useful WEB resources for *Salmonella* -omics**.

http://gsc.jcvi.org/projects/msc/salmonella/index.shtml	Genomic sequencing center for infectious disease (J. Craig Venter institute) *Salmonella* genome project (many serovar genome sequences, good resource)
http://www.ncbi.nlm.nih.gov/pmc/articles/PMC3032673/	*Salmonella* community effort metabolic model (Thiele et al., 2011) (down load model)
GSE32995	GEO genome array data sets, examples: GSE27703 Analysis of the host microRNA response to *Salmonella* uncovers the control of major cytokines by the let-7 family (Schulte et al., 2011) Transcriptional profiling of four growth phases *S*. Typhimurium comparing immobilized growth with planktonic growth
http://patricbrc.org/portal/portal/patric/GenomeList?cType=taxon&cId=590&dataSource=&displayMode=genome	Pathosystems Resource Integration Center (PATRIC) *Salmonella* genomes and large collection of sequences
*Salmonella*—Cbcb—umiacs https://wiki.umiacs.umd.edu/cbcb/index.php/Salmonella	wiki on *Salmonella* genome reads
http://www.poultryhub.org/production/food-safety/salmonella/	Poultry Hub (professional resource on *Salmonella* infections in veterinary medicine)
http://microbes.ucsc.edu/cgi-bin/hgGateway?hgsid=555757&clade=eukaryota-protista&org=Salmonella+typhimurium+LT2&db=0	Complete browsable genome viewer and genome sequence of *Salmonella enterica* serovar Typhimurium LT2 at UC Southern California
http://webclu.bio.wzw.tum.de/binfo/proj/proamp/Target_organisms/target_organisms.html	Integral membrane protein analysis of *Salmonella* (and other bacteria) at TU Munich
http://www.about-salmonella.com/	*Salmonella* food poisoning and outbreaks

#### Integrated analysis

Integration of high dimensional “-omics” datasets improves genome annotations, discovers novel virulence-related factors, and models *Salmonella* growth under infectious states (Ansong et al., 2012).

A multi-omics view on *Salmonella* in intestinal infection helps to better understand the interdependence of regulation and virulence vs. metabolic change, specific techniques and examples are given in Table 2. Thus, proteome, metabolome, glycome, and metagenome all change during the murine infection by *S. enterica* serovar Typhimurium. After multiplication in the mouse gut inflammation occurs and the whole microbiome changes: Bacteroidetes and Firmicutes are suppressed, *Salmonella* and *Enterococcus* grow (Deatherage Kaiser et al., 2013). In response to *S. enterica* serovar Typhimurium infection, potential novel innate immune factors can be discovered, there is transmigration and activation of neutrophils and up-regulation of cell surface molecules. Coordinate murine immune responses include complement activation and inflammatory antibacterial response. *Salmonella* metabolism reacts by induction of stress response proteins, synthesis of outer membrane proteins and lipoproteins.

**Table 2:** **Techniques to model *Salmonella* metabolism and its regulation**.

**Model**	**Insight**	**Author, weblink**
**TECHNIQUES TO STUDY METABOLIC ADAPTATION IN *SALMONELLA***		
*S. Typhimurium metabolite profiling for different (nutrient poor, virulence induced) environments and genome-scale metabolite model*	→Central carbon metabolism strongly altered (depletion in glycerol catabolism (glycerol, glycerol 3-phosphate, dihydroxyacetone phosphate, and pyruvate), increased glucose. Synthesis and uptake of polyamines (may protect *Salmonella* against osmotic stress inside host cells).	Kim et al., 2013
		doi: 10.1039/C3MB25598K
*Metabolic model and microbiology, metabolite measurements; for survival (unrelated to growth): “maintenance requirements” and “costs” (to resist host) are calculated as ATP expenditure*.	→ Accumulation of metabolites in the infected gut (lactose, galactinol, melibiose, and raffinose) *Salmonella* and murine host lack necessary enzymes → used by Bacteroidetes and other commensals (glycosidases).	Deatherage Kaiser et al., 2013
		PMID: 22168414
	→ Model predicted hundreds of virulence phenotypes with 90% accuracy.	
	→ Costs become very high under excess nutrient availability	
**Model**	**Result**	**Author, weblink**
**METABOLIC MODELING TECHNIQUES**		
*Hypothesis of nutrient-limitation during infection*	→ Inactivation of *Salmonella* enzyme → metabolic bottleneck → overexpression of another enzyme.	Steeb et al., 2013
		PMID: 23633950
*Deletion effects, e.g., calculations for* Δ*ppc*	→ No compensatory flux via the glyoxylate shunt.	Fong et al., 2013
		PMID: 23432746
**Model**	**Result**	**Author, weblink**
**MODELING REGULATION OF *SALMONELLA* METABOLISM**		
*Boolean modeling of genes in SPI1, SPI2, and T6SS Integrated: osmolarity, glucose, iron, calcium and magnesium concentrations, growth phase-dependent stationary phase factors*.	→ Description of pathogenicity island cross-talk (e.g., SPI2-secreted proteins low → activation of T6SS). → Antagonistic cross-talk (e.g., *SsrAB* to *SciS; MviA* to *RcsB*).	Das et al. (2013)
		doi: 10.1186/1757-4749-5-28
*Spatiotemporal distribution of ROS in neutrophils, macrophages carrying Salmonella and in vivo expression of ROS defense enzymes (KatG, SodAB, and host NADPH oxidase)*.	→ (a) Neutrophils: lethal concentrations of hydrogen peroxide	Burton et al. (2014)
	→ (b) Macrophages: only sub-lethal ROS concentration during infection	PMID: 24439899

**Figure 1:**
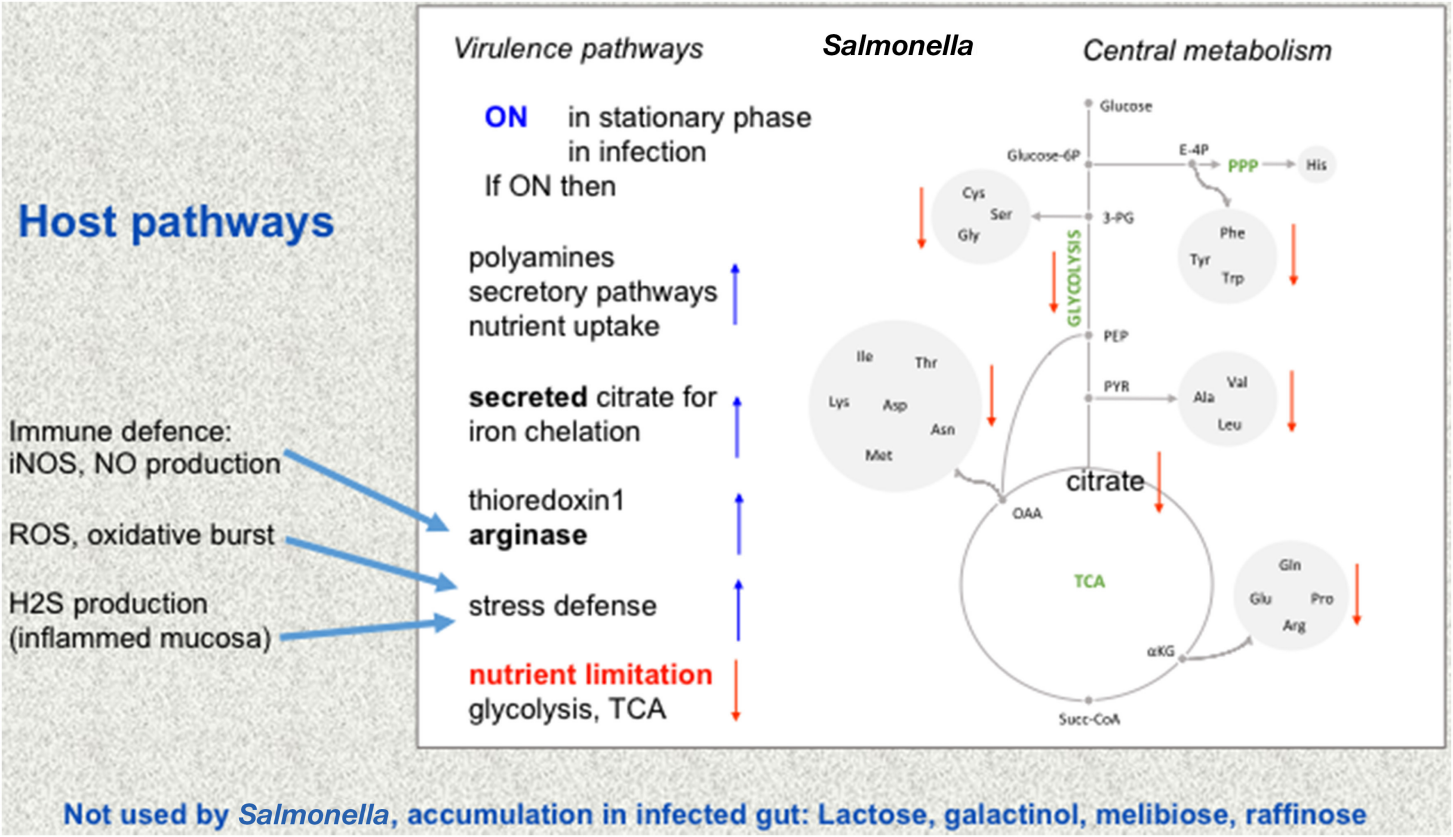
**Metabolic adaptation of *Salmonella***. Changes from intestinal to intracellular lifestyle in the mucosa and the resulting adaptations are depicted. The central metabolism of *Salmonella* is shown in the mid panel, while virulence pathways are shown on the left. Environments may change from rather nutrient-rich conditions to nutrient-restricted conditions (red letters) such as in infection, for instance when *Salmonella* is ingested with contaminated food. Amino acids are abbreviated by their three letter code. Other abbreviations: PPP, pentose phosphate cycle; E-4P, erythrose 4-phosphate; Glucose-6P, glucose 6-phosphate; TCA, tricarbonic acid cycle (citric acid cycle). Some of the ensuing changes in *Salmonella* pathways are indicated (right, blue arrows up or red arrows down compared to rich nutrient environment, e.g., TCA goes down while some of the now less used citrate is used to chelate iron). Some metabolic changes from the host that influence *Salmonella* metabolism (Winter et al., 2010; blue arrows) are given on the left. Bottom: these sugars which are not used by *Salmonella* nor by the host accumulate in the infected gut.

The combination of integrated analysis of the different data sets shows that *Salmonella* reshapes its metabolism for its adaptation to different host environments. Virulence-associated remodeling adapts *Salmonella* to new niches and locations in the host, there nutrient-poor conditions are encountered and a strong protection against hostile environments of the host is mounted (Figure 1).

#### Metabolic modeling

Metabolic modeling of *Salmonella* in infection reveals an integrated picture of *Salmonella* adaptation processes. Furthermore, in the past few years, several groups established extensive, well-curated, models of *Salmonella* metabolism (Raghunathan et al., 2009; Thiele et al., 2011). Metabolic models are refined by considering additional energy required for stress defense mechanisms and adaptation during infection (Steeb et al., 2013) or considering metabolic bottlenecks (Table 2).

#### Modeling regulation of salmonella metabolism

Several studies analyzed *Salmonella* regulatory networks of genes in various SPI by means of mathematical models (Temme et al., 2008; Bailly-Bechet et al., 2011). Current results allow to model close to observation the sequential activation of virulence gene clusters in adaptation to distinct host environments (Table 2).

In the analysis of *Salmonella*-human interactions, large-scale cellular networks can already be described by looking at their structure, without attempting a dynamical simulation. Such graph-based methods mainly focusing on the topology to predict the chain of events in signaling or estimate metabolic capabilities. Here, cellular modules for different functions are identified as sub-graphs (sub-networks) with proteins mediating only this function in the complete network. Furthermore, hubs, central nodes in the network receiving many connections and indicating strongly connected genes or proteins, are of interest. For instance, interactome networks describing protein-protein interactions are built up and serve as scaffolds for further analysis (Schleker et al., 2012).

Rosenkrantz et al. (2013) compared two types of networks for *S*. Typhimurium strain LT2 regarding stress response and metabolic adaptation: a transcriptional data network using transcriptional data for 425 selected genes under different growth and stress conditions identifying the significantly and strongly regulated genes (transcriptional network) for each condition. This was compared to a genome-scale network connecting genes with metabolic pathways and cellular functions. Looking at the top five connecting hub proteins from the transcriptional network (*wraB, ygaU, uspA, cbpA, and osmC*) as well as the hubs in the genome scale metabolic pathway and cellular function network (*ychN, siiF, yajD, ybeB, and dcoC*), all these hubs were found to be dispensable for virulence in mutation studies. However, double mutants of these two sets of regulatory proteins showed clear effects on virulence in mouse infection experiments (Rosenkrantz et al., 2013). This is a particular strong example confirming the robust and well-buffered *Salmonella* regulation of metabolism and cellular function with virulence factors having partly redundant, overlapping functions.

### Metabolic adaptation of *Salmonella* during stress conditions

#### Stress factors linking virulence and metabolism

**Figure 2:**
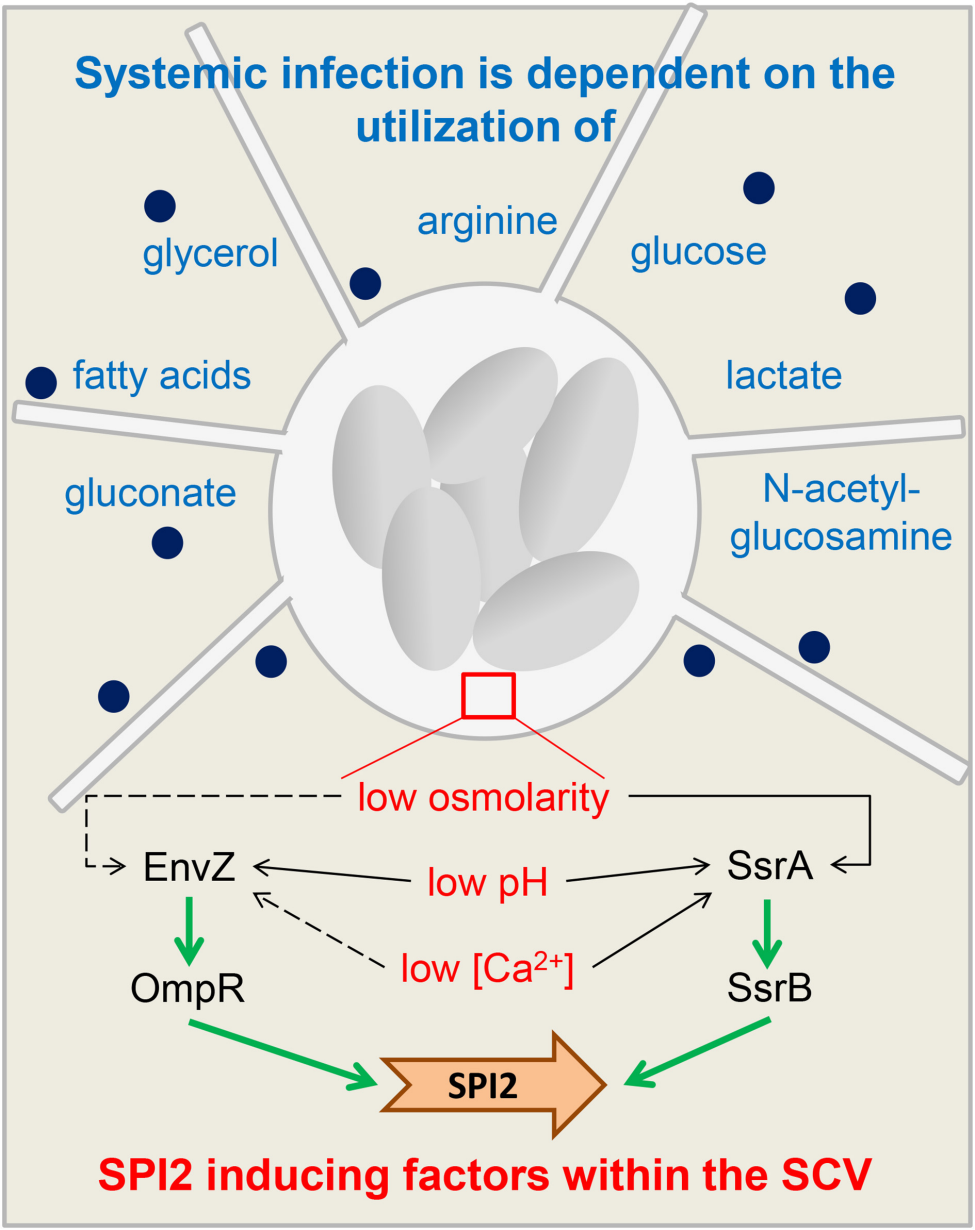
**Regulatory adaptations and nutritional requirements of *Salmonella***. A schematic overview of functions of SPI2 during host cell infection and nutritional requirements to manifest a systemic infection. The *Salmonella*-containing vacuole (SCV) with replicating *Salmonella* (gray ovals) is connected to *Salmonella*-induced filaments. Blue writing: substrates *Salmonella* depends on to manifest systemic infections (Steeb et al., 2013). Dark blue spots: SPI2 effectors. The insert (brown rectangle) shows key factors influencing SPI2 expression (Haraga et al., 2008). Detection of low osmolarity, low calcium concentrations and acidic pH by the two-component systems Envz/OmpR and SsrAB inside the SCV leads to activation of SPI2 gene expression (Garmendia et al., 2003). Dotted arrow: minor effect on EnvZ/OmpR activity and SsrAB activity. Non-dotted arrow: strong effect on EnvZ/OmpR activity and SsrAB activity.

When *Salmonella* enters into an intestinal epithelial cell, environmental factors such as high osmolarity and neutral pH lead to an activation of *HilD*, which in turn induces *HilA* and *invF* gene expression (Altier, 2005). *HilA* as transcriptional regulator in turn activates all SPI1 genes necessary for assembly of the T3SS (Ellermeier and Slauch, 2007) and translocation of various SPI1 effector and host interaction proteins (Sop proteins, SipA) as well as DksA to coordinate NAD(P)H/NAD(P)(+) redox balance under nutrient limitation (Henard et al., 2010). For instance, SopB protein changes host cell exocytosis (Perret and Zhou, 2013). SPI1 gene expression is dependent on the growth phase (e.g., there is highest SPI1 induction after 3.5 h of growth in rich medium, Cossart and Sansonetti, 2004). Effector protein activity leads to reorganization of the host cell actin cytoskeleton, followed by membrane ruffling and internalization of *Salmonella* (Haraga et al., 2008). Next key factors influencing SPI2 expression (Haraga et al., 2008) such as detection of low osmolarity, low calcium concentrations and acidic pH by the two-component systems Envz/OmpR and SsrAB lead to activation of SPI2 gene expression (Garmendia et al., 2003) with factors such as SifA, SseJ, PipB2, and SseG (Núñez-Hernández et al., 2014) and result in a SCV containing multiplying *Salmonella* and inducing filaments. The combined action of these regulatory mechanisms ensures that sufficient nutrients are available for *Salmonella* during the infection (Figure 2).

#### Metabolic defense pathways

*Salmonella* has to adapt its metabolism to different environmental stresses and niches when entering the human host, starting with the challenging acidic environment of the stomach (Table 3). Furthermore, immune defense reactions from the host involve free radicals, complement reaction, enzymatic degradation and autophagy reactions. Individual examples for these biochemical assaults on *Salmonella* have been studied in detail. Nitric oxide (NO) produced by the NO synthase of several immune cells of the host has a severe impact on central carbon metabolism of *Salmonella*. NO targets the pyruvate and α-ketoglutarate dehydrogenase complexes (Richardson et al., 2011).

**Table 3:** **Studies on different metabolic conditions for *Salmonella***.

**Condition**	**Result**	**Author, weblink**
**METABOLIC DEFENSE, NO**		
*NO (murine host) → lipoamide dehydrogenase (Salmonella) reduced activity*	→ Methionine and lysine precursor succinate low → transporters (e.g., for succinate) important under nitrosative stress	Richardson et al., 2011
		PMID: 21767810
*NO (murine host) → reduction of aerobic energy by nitrosylating terminal quinol cytochrome oxidases*.	→ Diminishes energy-dependent aminoglycoside uptake → protects antibiotic challenges during host nitric oxide generation	Husain et al., 2008
		PMID: 18198179
		McCollister et al., 2011
		doi: 10.1128/AAC.01203-10
*NO (murine host) → decrease in NADH dehydrogenase activity → NADH high in cytoplasm hydrogen peroxide protection*	→ Direct detoxification of NO by the NADH dehydrogenase (RNS defense by acid-induced regulator Fur regulates NADH dehydrogenase)	Husain et al., 2008
		PMID: 18198179
		Husain et al., 2014
		PMID: 24166960
**Condition**	**Result**	**Author, weblink**
**CARBOHYDRATE METABOLISM**		
*Carbohydrate metabolism adaptations of Salmonella during infection*	→ Aconitase isoenzymes: acoA for oxidative stress	Baothman et al., 2013
	→ Repair of oxidized aconitase by bacterial frataxin ortholog proteins CyaY and YggX	PMID: 23637460
		Velayudhan et al., 2014
		PMID: 24421039
*S. Typhimurium TCA cycle mutations (gltA, mdh, sdhCDAB, sucAB, and sucCD*	→ Incomplete TCA helps survival and replication in resting and activated murine macrophages compared to wt	Bowden et al., 2010
	→ Epithelial cell infection: Δ*sucCD* and Δ*gltA* replicate less than wt	doi: 10.1371/journal.pone.0013871
	→ *S*. Typhimurium Δ*sucAB* and Δ*sucCD* attenuated in murine infection	
**Influence**	**Result**	**Author, weblink**
**THE BROAD INFLUENCE OF AMINO ACIDS ON METABOLIC ADAPTATION DURING INFECTION**		
*Amino acid decarboxylase systems consume protons, raise cytosolic pH*	→ *Salmonella* decarboxylases for lysine (*CadA*), arginine (*AdiA*), and ornithine (*SpeF*), not glutamate → acid tolerance but not essential for virulence in mice	Alvarez-Ordóñez et al., 2010
		PMID: 19864032
		Viala et al., 2011
		PMID: 21799843
*Arginine has no decarboxylase, but key immune modulator from Salmonella*	→ Substrate competition *Salmonella* arginase II and iNOS of the host	Das et al., 2010
		doi: 10.1371/journal.pone.0015466
	→ *Salmonell*a up-regulates arginase II activity in RAW264.7 macrophages → down regulates host iNOS → by this in intestinal lumen beneficial increase of electron acceptor nitrate	Lahiri et al., 2008
		PMID: 18625332
		Humphreys et al., 2012
		PMID: 22341462
**Feature**	**Result**	**Author, weblink**
**THE INTERPLAY OF *SALMONELLA* PATHOGENICITY ISLANDS AND METABOLISM**		
*IacP downstream of sipA for effector protein within SPI1*	→ Facilitates *Salmonella* invasion to HeLa cells by secretion of SPI1 effectors *SopA, SopB*, and *SopD*	Kaniga et al., 1995
		PMCID: PMC177584
	→ *IacP* activated by 4′-phosphopantetheine transferase AcpS	Kim et al., 2011
		PMID: 21263021
		Viala et al., 2013
		PMID: 23893113
*The transferase is a link between bacterial fatty acid metabolism and SPI1 virulence (Hung et al., 2013)*	→ Propionyl-CoA represses *AcpS*, *HilD*, *Salmonella* invasion	Hung et al., 2013
	→ Low SopB secretion	PMID: 23289537
	→ May be priming of fatty acid metabolism inside the SCV.	Viala et al., 2013
		PMID: 23893113
*Virulence → high iNOS, and NO levels → radical chain reaction, isomeri-zation → nitrate increase*	→ Growth advantages for nitrate respiring strains such as SL1344	Lopez et al., 2012
		PMID: 22691391
*In SCV nitrate respiration tries to avoid host cell damage*	→ *NapA* respiration instead of *NarG* pathway (Rowley et al., 2012)	Rowley et al., 2012
		PMID: 22039967
**INTRACELLULAR ADAPTATION AND METABOLISM OF *SALMONELLA***		
*polyamines required for replication in epithelial cells (Jelsbak et al., 2012)*	Δ*spe* polyamine synthesis mutant → no polyamines → decreased invasion ability (*hilA* lower → *invF* and *sipB* virulence factor down)	Jelsbak et al., 2012
		PMID: 24602405
*Glycerol and glucose → major carbon sources in systemic infections (Eisenreich et al., 2013)*	→ Δ*tpi* triose phosphate isomerase mutant attenuated in mouse infection → Δ*glpE* mutant strain, too	Eisenreich et al., 2013
		PMID: 23847769
		Paterson et al., 2009
		PMID: 19493007
*Amino acid starvation in host → xenophagy, autophagy dependent targeting and degradation of intracellular bacteria*	→ Requires host mTOR pathway triggered by autophagy-related gene (ATG) protein 13	Tattoli et al., 2012
		PMID: 22704617
		Ganley et al., 2009
		PMID: 19258318
		Kamada et al., 2000
		PMID: 10995454

#### Carbohydrate metabolism

Citrate is a TCA cycle intermediate (Figure 1) and is an important regulatory molecule in the control of glycolysis and lipid metabolism (Neidhardt, 1996). Furthermore, acetylation and deacetylation regulate the amount of glycolysis vs. gluconeogenesis as well as branching between citrate cycle and glyoxylate (Wang et al., 2010; Table 3). Moreover, citrate is a crucial iron-chelator which is involved in the homeostasis of iron in the pathogen, as well as the host. Iron is an essential component for several enzymes, but in high concentrations, it may cause damage. Citrate is consumed during NO exposure and other stress conditions because the export pump IctE (iron citrate efflux transporter, former called MdtD) transports iron chelated with citrate out of the cell. Export of citrate leads to growth arrest (Frawley et al., 2013), a status that allows it to survive antibiotic challenges as observed in persister bacteria. This function decreases harmful cellular iron content and reduces growth of *Salmonella* making it more stress resistant (Figure 1).

***The broad influence of amino acids on metabolic adaptation during infection***. The work on acetylation regulation in *Salmonella* by Wang et al. (2010) also underlines also that the central carbon as well as connected amino acid metabolism, including the TCA cycle, can directly be linked to stress response (Figure 1).

In particular, the bacterial arginine permease *ArgT* is an essential virulence determinant which decreases the host's cellular arginine content and reduces by this way the NO production of the host (Das et al., 2010; Table 3). In contrast, arginine degradation by *Salmonella* appears to be without influence on NO production. Although arginine degradation pathways are up-regulated in *Salmonella* during infection of macrophage and essential for virulence, this is due to other mechanisms but not related to substrate degradation of iNOS (Choi et al., 2012).

Cysteine is a key amino acid during oxidative stress response in *Salmonella*. In a study on cysteine biosynthesis during oxidative stress, cysteine biosynthesis regulation was blocked in Δ*cysB* and Δ*cysE* mutants and oxidative defense pathways encoded by *katG* and *soxS* were up-regulated compared to the wild-type strain (Turnbull and Surette, 2010). Consequently, the cysteine biosynthesis and cysteine-derived molecules such as thioredoxin play an important role for intracellular *Salmonella* survival and replication (Bjur et al., 2006). In this regard, the oxidoreductase thioredoxin 1 (TrxA) was found to be co-induced and essential for SPI2-T3SS activity under conditions that mimic life in the SCV (Negrea et al., 2009).

### The richness of *Salmonella* metabolism and its influence on virulence

The *SsrAB* virulon controlling SPI2 gene expression is induced under nutrient-poor conditions (e.g., presence in the phagosome, Kuhle and Hensel, 2004).

#### The interplay of salmonella pathogenicity islands and metabolism

Various metabolic pathways which have an impact on the SPI1 activity of *Salmonella enterica* (Table 3). One example is the interaction between the invasion acyl carrier protein (IacP; Viala et al., 2013) and secretion of SPI1 effector proteins into the host cell to achieve rearrangement of the host cytoskeleton and engulfment of the bacterium (reviewed in Cossart and Sansonetti, 2004).

There are also indications for the influence of SPI1 functions on the host's metabolism in order to facilitate survival in the intestine and subsequently intracellular to promote the infection process. Thus, the SPI1-T3SS effector protein SopE is known to increase *Salmonella* invasiveness and to induce strong inflammatory host responses (Humphreys et al., 2012).

Although SPI1 and SPI2 are induced under very distinct nutritional environments (SPI1 in a nutrient rich environment, SPI2 by nutrient starvation, Kuhle and Hensel, 2004), there are some bacterial metabolites which effect SPI1 as well as SPI2 activity and have a general impact on *Salmonella* virulence (Table 3). One example are polyamines, short cationic amines, of which spermidine and putrescine are mostly common in bacteria (Jelsbak et al., 2012).

***Intracellular adaptation and metabolism of Salmonella***. While conditions in the intestinal lumen are nutrient rich, the situation changes after *Salmonella* invades into the epithelial cells and is phagocytosed at the basolateral cell side by macrophages or dendritic cells. Staying inside the SCV, the pathogen has to deal with nutrient limitations. To investigate which metabolites could interact with expression of genes in SPI1 or mainly SPI2, one issue is to define the nutritional situation of *Salmonella* gain inside the SCV and to figure out which metabolites *Salmonella* has access to. Mouse infection experiments showed on the one hand that intracellular *Salmonella* get access to a wide range of nutrients, including nearly all amino acids except proline. On the other hand, it was shown that the ability to manifest a full systemic infection is dependent on the utilization of “glycerol, fatty acids, N-acetylglucosamine, gluconate, glucose, lactate, and arginine” (Steeb et al., 2013). However, *Salmonella* is able to counteract various defense mechanisms in order to facilitate growth or reduce immune responses (Table 3). Invasion of pathogens into epithelial cells is followed by cytosolic amino acid starvation in host cells, which seems to be explained by membrane damage during the invasion process (Tattoli et al., 2012).

However, in contrast to *Shigella*-infected cells, amino acid levels of epithelial cells invaded by *Salmonella* normalized 3 h after infection, which leads to relocalization of mTor invasion sustaining pathway to the SCV, phosphorylation of ATG protein 13, leading to a low ATG protein 1 activity and thus reduced autophagy (Ganley et al., 2009). By this, *Salmonella* is able to avoid autophagy in epithelial cells. Further investigations are required to clarify if normalization of amino acid levels is directly induced by *Salmonella*. At least the invasion-induced membrane disturbance is only severe in the first hour of infection and somehow repaired faster than in cases of invasion by other intracellular pathogens (Tattoli et al., 2012).

### Anti-infective strategies in the face of robust *Salmonella* metabolism

As *Salmonella* adapts rapidly and successfully to changing conditions including intracellular survival in macrophages, in epithelia and in the gut, we will now examine which antibiotic strategies are nevertheless available for *Salmonella* infections. A seminal work by Becker and co-workers showed that the robust metabolism of *Salmonella* limits possibilities for new antibiotics (Becker et al., 2006) and Bumann stressed this point asking “has nature already identified all useful antibacterial targets?” (Bumann, 2008). It is of course important to mention the billions of years sampling time to test and select bacteria and bacterial metabolism during evolution. Furthermore, the parallel exploitation of diverse host nutrients often enhances often *Salmonella* virulence (Steeb et al., 2013) and persistent *Salmonella* are highly resilient (Barat et al., 2012). On the other hand, as many medical areas such as cancer research or aging research also make clear, any medical intervention happened only very recently in evolutionary times. Hence, additional medical interventions are not limited by evolutionary constraints such as positive epistatic selection or direct metabolic energy costs. There are many potential targets still in stock, both by targeting metabolic pathways in pathogenic bacteria and *Salmonella* in particular, as well as by exploring novel ways of anti-infectives. One inspiring example is metabolic engineering of *Salmonella* vaccine bacteria in the mevalonate pathway to boost human Vγ2Vδ2 T cell immunity (Workalemahu et al., 2014). As reviewed earlier (Dandekar and Dandekar, 2010), anti-infective action starts furthermore from typical hygienic measures such as isolation of patients with multi-resistant strains including silent clinical carriers, but also includes targeted disturbance of metabolic pathways for example by sulfonamides. In particular, both targeted therapy (direct delivery of an antibiotic to only the location it should act, e.g., in the intestine) as well as targeted modification of standard drugs (so that they are more detrimental to the pathogen even if the host shares similar proteins) are options which have high potential and are not much explored. Our own research highlights the interconnectivity of metabolism. This renders *Salmonella* also vulnerable also in conserved and well investigated pathways, such as TCA cycle and its anaplerotic reactions. Thus, *Salmonella* Typhimurium is controlled by host NO production as shown in mice experiments *in vivo*. Methionine or lysine auxotrophy results from reduced succinyl-CoA availability as the lipoamide dehydrogenase activity is targeted by NO while compensatory *Salmonella* pathways to achieve more succinyl-CoA are again blocked by NO (Richardson et al., 2011). Here it also becomes obvious why the therapeutic strategies are not easy exhausted, for instance by direct delivery of NO-increasing drugs to the severely infected gut. Anesthetic drugs are membrane modifiers yielding even multi-resistant pathogens again vulnerable to additional antibiotics, just to cite another possibility (Dandekar and Dandekar, 2010). Furthermore, novel vaccination strategies may proof successful. Hence, the task is more to implement some of the many open alleys for novel antibiotic therapies in clinic. This includes targeting of the metabolism. Furthermore, clinical studies are required for each novel antibiotic strategy, these are currently too expensive for high patient numbers and the prize margin for antibiotics is low so antibiotic development pipelines dry out. However, the prices for such clinical studies could be drastically lowered by modern patient hospital information systems, and furthermore, public awareness and willingness to have better protection against infections is currently increasing.

### Conclusions: *Salmonella* general metabolic lifestyle during infection

We saw that multiple “-omics” and especially metabolomic data are currently used to determine the needs for *Salmonella* to facilitate intracellular survival within the SCV in host cells and its nutrient supply.

*Salmonella's* generalist metabolic lifestyle meets all types of environmental challenges, be it ROS or nutrient limitation by its broad metabolic capabilities. The broad metabolism suggests nevertheless potential for novel anti-infective strategies. However, under severe conditions *Salmonella* regulation and metabolism are spiked up by input from SPI1, SPI2, T3SS and T6SS, modified invasion abilities, redox protection and central metabolism to turn the neutral environmental lifestyle of *Salmonella* into a pathogenic lifestyle for its host. On top of this such genetic modules catalyze rapid genetic exchange between *Salmonella* strains showing that only an integrated picture will help to sustain antibiotic efficiency against *Salmonella* infections.

### Conflict of interest statement

The authors declare that the research was conducted in the absence of any commercial or financial relationships that could be construed as a potential conflict of interest.
